# Influence of Grapevine Cultivar on the Second Generations of *Lobesia botrana* and *Eupoecilia ambiguella*

**DOI:** 10.3390/insects9010008

**Published:** 2018-01-19

**Authors:** Francesco Pavan, Giorgio Stefanelli, Alberto Villani, Elena Cargnus

**Affiliations:** 1 Department of Agricultural, Food, Environmental and Animal Sciences, University of Udine, Via delle Scienze 206, 3100 Udine, Italy; elena.cargnus@uniud.it; 2 Agenzia Regionale per la Protezione dell’Ambiente del Friuli Venezia Giulia, Dipartimento di Udine, via Colugna 42, 33100 Udine, Italy; giorgio.stefanelli@arpa.fvg.it; 3 Agenzia Regionale per la Protezione dell’Ambiente del Friuli Venezia Giulia, OSMER, via Natisone 43, 33057 Palmanova (UD), Italy; alberto.villani@arpa.fvg.it

**Keywords:** European grapevine moth, vine moth, cultivar susceptibility, northeastern Italy, larval infestation, larval phenology

## Abstract

Grapevine cultivar can affect susceptibility to *Lobesia botrana* and *Eupoecilia ambiguella* with important implications on control strategies. A four-year study was carried out in north-eastern Italy on 10 cultivars (Cabernet Sauvignon, Carménère, Chardonnay, Merlot, Refosco dal Peduncolo Rosso, Rhine Riesling, Sauvignon Blanc, Terrano, Tocai Friulano and Verduzzo Friulano) grown in the same vineyard to assess whether the cultivar affects second-generation population levels of the two vine moths and *L. botrana* larval age composition. The influence of bunch traits measured at the peak of egg hatching on demographic parameters was also evaluated. Over the four years, *L. botrana* significantly prevailed over *E. ambiguella* in nine cultivars. Chardonnay and Tocai Friulano were the most infested cultivars and Merlot was the least infested. At the sampling date, the age composition of *L. botrana* varied with cultivar, with the larvae being significantly older on Chardonnay and younger on Carménère, Merlot and Verduzzo Friulano. Older larval age was significantly associated with higher bunch compactness. Larval infestation was not significantly influenced by either bunch compactness or berry volume, which suggested a more important role for contact and volatile substances mostly originating from the berries. These results allow for the improvement of Integrated Pest Management strategy against vine moths.

## 1. Introduction

The European grapevine moth, *Lobesia botrana* (Denis & Shiffermüller) (Lepidoptera Tortricidae), and the vine moth, *Eupoecilia ambiguella* (Hübner) (Lepidoptera Tortricidae), are the principal insect pests in European vineyards. In northeastern Italy, the two species have two or three generations, depending on different microclimatic conditions [1]. The *Vitis vinifera* L. cultivar can influence susceptibility and sensitivity to *L. botrana* and *E. ambiguella* [2,3,4,5,6,7,8,9,10,11,12,13,14,15,16,17,18,19,20,21,22,23,24,25,26,27,28] as well as to other grapevine insect pests (e.g., leafhoppers [29,30] and scales [31]).

Different cultivar susceptibilities, expressed as moth larval infestation, have been reported in reviews [2,3] and experimental studies [4,5,6,7,8,9,10,11,12]. For *L. botrana*, they have been associated with egg-laying preference [4,13,14] and greater larval settlement and survival [15,16,17]. The cultivar could also affect moth infestation levels through an influence on abundance and diversity of larval parasitoids [18].

Differences in *L. botrana* performance due to cultivar have been observed in the laboratory for larval development time, fecundity, egg size and egg hatchability [19,20]. In the field, some cultivars have been associated with earlier larval development during the first generation [7] and others with both higher pupal weight and earlier adult emergence during the second generation [21]. 

Some research has investigated the factors involved in cultivar susceptibility to *L. botrana* infestation. For the first generation, infestation levels were positively correlated with inflorescence earliness [2,5,7] and shorter duration of blooming [2,15] and negatively correlated with inflorescence hairiness [2,7]. Although inflorescence compactness has been reported to be favourable to the anthophagous generation [2,5], its role seemed less important than other factors [7]. Lower inflorescence hairiness has been associated with an earlier development of *L. botrana* larvae, probably because females start to lay eggs earlier on smooth inflorescences [7]. For the carpophagous generations, in many studies, bunch compactness has been reported as the most important factor favouring cultivar susceptibility [2,3,6,9,10,11,12,17] and, in particular, it was shown that the mortality of newly-hatched *L. botrana* larvae is higher on loose bunches during both the second and third generations [17]. Based on laboratory and field studies, the different susceptibility of cultivars has been associated with contact or volatile substances from berries that stimulate females to lay more eggs on some cultivars [4,14,22,23]. Some early-harvest cultivars are more infested by the second generation but less by the third generation when compared to late-harvest cultivars [11,24]. This is probably because females of the second generation laid few eggs on cultivars close to ripening [25].

The same larval infestation can be associated with different amounts of damage due to variability in cultivar sensitivity. During the first generation, larval feeding on the same number of flowers can cause a reduction in berry number in some cultivars but not others [26]. During the second generation, the cultivar influences the number of berries damaged by a single larva and the spread of bunch rots from bored berries to the contiguous non-bored ones [8,27]. The yield losses caused by second-generation larvae are higher on early harvest cultivars than on late harvest cultivars because, on the latter, the spread of bunch rots from bored to contiguous non-bored berries is lower and these healthy berries can offset the weight reduction of the bored berries [28].

The aims of the present study were: (i) to compare the population levels of *L. botrana* and *E. ambiguella* during the second generation on 10 grapevine cultivars grown in a northeastern Italian vineyard; (ii) to verify whether the two moths have different cultivar preferences; (iii) to verify whether *L. botrana* larval phenology (i.e., larval age composition) is influenced by the cultivar in agreement with adult emergence differences reported in literature [21]; (iv) to verify whether certain bunch traits can explain differences among cultivars regarding susceptibility and larval phenology; and (v) to verify whether any correlation exists between population data collected during the first and second generations (data reported in [7] and in this study, respectively). In considering both moth species in two different generations and the higher numbers of cultivars than previous studies and our assessment of bunch traits, the present study provides novel knowledge in the understanding of the biology and management of *L. botrana* and *E. ambiguella*.

## 2. Materials and Methods

### 2.1. Sampled Vineyard

The study was carried out in a flatland vineyard of northeastern Italy (Friuli Venezia Giulia region, Pordenone district, Spilimbergo locality, 12°55’ E, 46°6’ N, 101 m altitude a.s.l.). The sampled vineyard was planted in 1980 and covers an area of about 1 ha with north–south oriented rows. The vineyard was bordered by a vineyard of the cultivar Sauvignon Blanc on the south side and surrounded by annual arable crops (i.e., maize and soybean) on the other three sides. The grapevines were Sylvoz trained and spaced 3.5 m between rows and 1.75 m along rows. Eleven cultivars, grafted to Kober 5BB, were grown: Cabernet Sauvignon, Carménère, Chardonnay, Merlot, Pinot Gris, Refosco dal Peduncolo Rosso (hereafter, Refosco p.r.), Rhine Riesling, Sauvignon Blanc, Terrano, Tocai Friulano, and Verduzzo Friulano). Each cultivar was replicated in two adjoining rows. The position of each cultivar within the vineyard was independent from this study. Green pruning, fertilization, irrigation and fungicide applications were the same across the vineyard. No insecticides were applied. In the area where the vineyard is located, *L. botrana* and *E. ambiguella* have two generations a year.

### 2.2. Larval Population Levels of the Two Vine Moths

During 1994–1997, the population levels of the second generation of the two vine moths were estimated in 10 out of the 11 cultivars. Our study did not consider Pinot Gris because, after fruit set, many grapevines showed physiological problems that stopped berry development.

To estimate the population level, 100 bunches per cultivar (50 per row) were sampled just before berries developed colour (beginning of August) when the earliest larvae had not yet started to pupate. The sampled bunches were chosen on the basis of a fixed scheme to avoid subjective choice [32]. On each bunch, the number of larval nests was counted and larvae found inside the nests were collected. To determine the relative proportion of the two vine moths on each cultivar, further bunches were sampled until either 50 larvae per cultivar were found or all bunches of each cultivar were checked. All the collected larvae were identified at species level in the laboratory under a dissecting microscope on the basis of head-capsule colour and body-cuticle granulation [33]. 

Repeated measures ANOVA and a Tukey’s test were used to compare larval infestations among different cultivars. Analyses were conducted for the two moth species individually and together. Because, in a few cases, larvae were not found inside larval nests, in order to allocate the total infestation level between the two moth species, for each cultivar, the total number of larval nests was multiplied by the relative species proportion among the collected larvae. A *G*-test was used to determine whether the relative proportion of the two moth species for each year and cultivar was significantly different from 50%. A *G*-test was also used to determine whether the relative proportion of the two species was significantly different on a year-to-year basis when compared to the average of the four years. To establish whether the two moth species infested the cultivars differently, the relative proportion of the two moth species on each cultivar was compared to that of the total population using a Cochran’s *Q*-test. 

### 2.3. Age Composition of *L. botrana* Larvae

In 1997, at the sampling time, the *L. botrana* larvae found inside the nests were taken to the laboratory and mounted on slides to determine the instar on the basis of mandible length (the left one) [1,34]. It was measured with a precision of 1.25 μm under a compound microscope at a magnification of 400× using a calibrated ocular micrometer. For the purpose of this study, it was established whether the larvae belonged to the three first instars (mandible length ranging from 60 to 135 μm) or the 4th to 6th instars (mandible length ranging from 140 to 315 μm). In the area where the vineyard is located, the second-generation larvae of *L. botrana* develop through six instars [1,34].

To determine whether the phenology of *L. botrana* larvae was affected by the grapevine cultivars, the proportion of 4th to 6th instar larvae on each cultivar was compared with all cultivars together using a *G*-test. Pearson’s correlation was used to establish whether larval age composition was significantly related to larval infestation.

### 2.4. Bunch Traits during *L. botrana* Second-Generation Egg Hatching 

In 2013, for all the cultivars except Terrano, some bunch traits (i.e., compactness, berry number, total and average berry volumes) were measured. Ten bunches per cultivar were collected at the peak of *L. botrana* egg hatching as expected on the basis of male captures in pheromone traps checked daily. The collection time was established according to the fact that cultivar influences larval settlement [17].

To estimate the bunch compactness of each cultivar, a graduated beaker (high 95 mm and inner diameter 70 mm) filled with water up to 220 mL (i.e., up to 2/3 of its height) was used. The bunches were immersed individually from the distal part until the apex touched the beaker bottom. The volume of water after bunch immersion and the length of the portion of the bunch immersed were recorded. Then, a compactness index was calculated by dividing the amount of increased volume of the water after bunch immersion by the length of the immersed portion.

To estimate the average berry volume, the berries on 10 bunches per cultivar were detached from the rachis and counted. Then, the total volume of berries was established by measuring the increase in water volume after immersing the berries into a graduated beaker. The average berry volume was then calculated by dividing the volume of berries by the number of berries.

ANOVA and a Tukey’s test were used to compare the measured traits and calculated parameters of the nine cultivars. To determine whether cultivar susceptibility and larval age composition for *L. botrana* were influenced by certain bunch traits, linear regressions were performed. In particular, both 4-year average larval nests on 100 bunches and the percentage of 4th–6th larval instars (dependent variables) were regressed on bunch traits (independent variables).

### 2.5. Correlation between First- and Second-Generation Demographic Parameters

Based on the data collected in the same years and cultivars for the *L. botrana* first [7] and second generations (this study), Pearson’s correlations were used to assess the significance of larval infestation, the moth species preference for different cultivars and the earliness of larval development between the two generations.

## 3. Results

### 3.1. Proportion of the Two Vine Moths on Larval Population 

**Figure 1:**
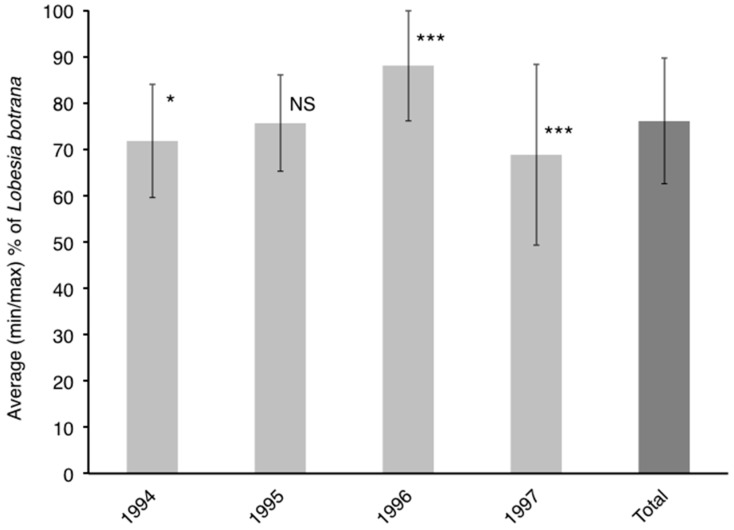
Proportion of *Lobesia botrana* larvae with respect to that of both moth species calculated for each year and all the years together (Total). *Eupoecilia ambiguella*% = 100% − *L. botrana*%. NS, * and *** indicate, respectively, not significant, significant differences at the 0.05 level, and significant differences at the 0.001 level between each year and Total, according to a *G*-test.

Considering all the cultivars together, the larval nests (average ± SD) per bunch during the four sampling years were 30.6 ± 20.0, 27.8 ± 19.1, 12.7 ± 8.8 and 24.4 ± 9.0. In all four years, a significantly higher proportion of *L. botrana* than *E. ambiguella* larvae was found (*p* < 0.01 according to *G*-test for all years). The proportion of *L. botrana* larvae for three years significantly differed from the total of all years combined (Figure 1). Specifically, it was significantly higher in 1996 and lower in 1994 and 1997.

### 3.2. Influence of Cultivar on Larval Population Levels

The average population levels over the four years significantly differed among cultivars considering the larval nests counted for the two moth species together (F_9, 27_ = 3.88, *p* = 0.003) and *L. botrana* (F_9, 27_ = 34.98, *p* = 0.0005), but not for *E. ambiguella* (F_9, 27_ = 1.97, *p* = 0.08) (Table 1). Considering both moths together or *L. botrana* alone, Chardonnay and Tocai Friulano were significantly more infested than at least one of the other cultivars. Carménère was the least infested cultivar. No evident gradient of infestation was observed within the vineyard. The population density exceeded the economic injury levels, i.e., 20 larval nests per 100 bunches [35], in all years for Chardonnay and Tocai Friulano, in three years for Sauvignon Blanc, Terrano and Verduzzo Friulano, in two years for Cabernet Sauvignon, Merlot and Refosco p.r. and in one year for Carménère and Rhine Riesling (data not reported).

On the four-year average, *L. botrana* prevailed over *E. ambiguella* with significant differences for all the cultivars, except for Carménère (0.05 level for Cabernet Sauvignon, 0.01 level for Merlot, and <0.001 level for the others according to a *G*-test). 

**Figure 2:**
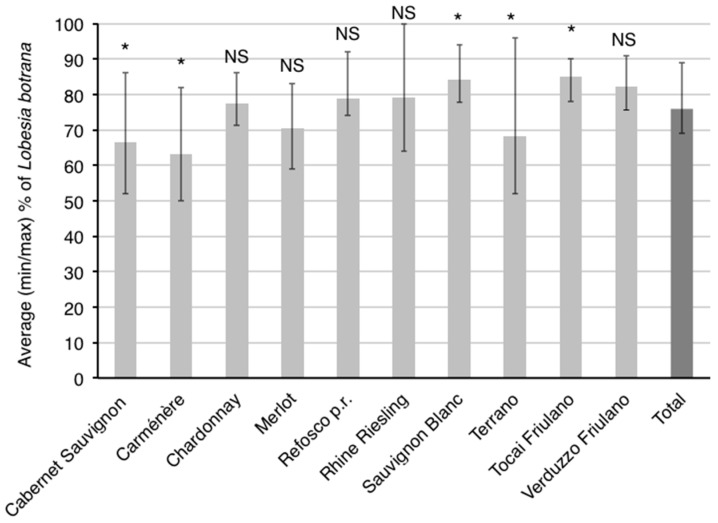
Proportion of *Lobesia botrana* larvae on the total of both moth species calculated for each cultivar and all the cultivars together (Total). *Eupoecilia ambiguella*% = 100% − *L. botrana*%. NS and * indicate, respectively, not significant and significant differences at the 0.05 level between each cultivar and Total, according to a Cochran’s *Q*-test.

On five cultivars, the relative proportion of *L. botrana* and *E. ambiguella* larvae (this last one calculated as: *E. ambiguella*% = 100% − *L. botrana*%) differed significantly from that calculated for all the cultivars together (Figure 2). In particular, the percentage of *L. botrana* larvae was significantly higher for Sauvignon Blanc and Tocai Friulano, and that of *E. ambiguella* for Cabernet Sauvignon, Carménère and Terrano.

### 3.3. Influence of Cultivar on Phenology of *L. botrana* Larvae

**Figure 3:**
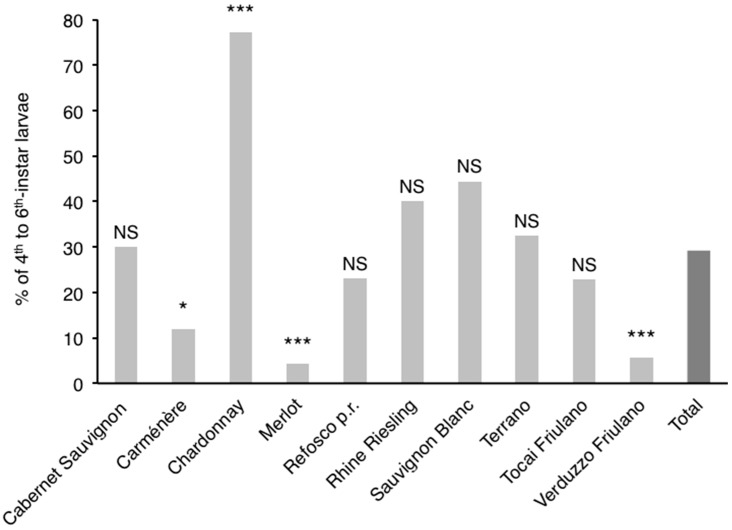
Percentage of 4th to 6th instar larvae of *Lobesia botrana* calculated for each cultivar and all cultivars together (Total). NS, * and *** indicate, respectively, not significant, significant differences at the 0.05 level and significant differences at the 0.001 level between each cultivar and Total, according to a *G*-test.

In 1997, the proportion of larvae belonging to the 4th to 6th instars was significantly different among cultivars (Figure 3). In particular, their proportions were significantly higher for Chardonnay and significantly lower for Carménère, Merlot and Verduzzo Friulano in comparison to the proportion calculated for all cultivars together.

There was no significant positive Pearson’s correlation between larval infestation and larval age composition (i.e., percentage of larvae of 4th–6th instars) (*p* = 0.21; *R* = 0.46).

### 3.4. Cultivar Bunch-Traits and *L. botrana* Demographic Parameters

The compactness index, measured at the peak of *L. botrana* egg hatching, was significantly different among cultivars (F_8, 81_ = 6.04; *p* < 0.0001) (Table 2). In particular, the lowest value was found in Merlot and the highest value in Chardonnay, with the other cultivars showing intermediate values.

The number of berries per bunch was significantly different among cultivars (F_8, 81_ = 7.59; *p* < 0.0001) (Table 2). The lowest values were found in Merlot, Carménère and Rhine Riesling, and the highest values in Refosco p.r., Sauvignon Blanc, Tocai Friulano, Cabernet Sauvignon and Verduzzo Friulano, with Chardonnay showing an intermediate value.

The total berry volume per bunch was significantly different among cultivars (F_8, 81_ = 4.63; *p* = 0.0001) (Table 2). In particular, the lowest values were found in Sauvignon Blanc, Rhine Riesling, Verduzzo Friulano and Merlot, and the highest value was found in Refosco p.r., with the other cultivars showing intermediate values.

The average berry volume was significantly different among cultivars (F_8, 81_ = 19.26; *p* < 0.0001) (Table 2). The lowest values were found in Sauvignon Blanc, Verduzzo Friulano and Tocai Friulano, and the highest values in Carménère, Chardonnay, Merlot and Refosco p.r., with the other cultivars showing intermediate values.

The larval infestation was not significantly influenced by the compactness index, the total berry volume per bunch, or the average berry volume (Table 3).

The percentage of 4th to 6th instar larvae was significantly influenced by the compactness index, but not by total berry volume per bunch or average berry volume (Table 3). 

### 3.5. Correlation between First- and Second-Generation Demographic Parameters

The Pearson’s correlation between the larval infestations in the first and second generations with reference to the total population across the four years was not significant (*p* = 0.60; *R* = 0.40).

The Pearson’s correlation between the larval infestations observed in each of the 10 cultivars in the first and second generations was not significant when considering either the total population (*p* = 0.73; *R* = 0.13) or the two moth species separately (for *L. botrana*: *p* = 0.68; *R* = 0.15; for *E. ambiguella*: *p* = 0.93; *R* = −0.03).

The Pearson’s correlation between the percentages of *L. botrana* in the total population of the first and second generations in the same year was not significant (*p* = 0.17; *R* = 0.47).

The Pearson’s correlation between the earliness in larval development recorded in each of the 10 cultivars in the first and second generations was not significant (*p* = 0.54; *R* = 0.22).

## 4. Discussion

### 4.1. Influence of Cultivar on Second-Generation Population Levels of the Two Vine Moths

The grapevine cultivar influenced both the larval infestation levels and the proportion of the two vine moths. In particular, Chardonnay and Tocai Friulano were the most infested and Carménère the least infested. The most infested cultivars were the same both considering the two moth species together and *L. botrana* alone. The higher susceptibility of Chardonnay and Tocai Friulano in comparison to Merlot and Cabernet Sauvignon is in agreement with other studies [8,11]. Knowledge of cultivar susceptibility to *L. botrana* infestation has important implications for Integrated Pest Management in the investigated grape-growing area, because the sampling time to establish whether the action threshold is exceeded can be considerably reduced. In multi-cultivar vineyards homogeneous for age, training system and cultivar practices, the following sequential procedure can be adopted: (1) the most susceptible cultivars should be sampled first; (2) the least susceptible cultivars, considering their decreasing relative infestation level risk, should be sampled only when the action threshold is exceeded in the former cultivars; and (3) sampling can be stopped when the action threshold is not exceeded in cultivars with higher relative infestation levels. This sampling procedure has been successfully adopted for the first generation of vine moths [7] and for *E. vitis* [29].

*L. botrana* showed a preference relative to *E. ambiguella* for the two which cultivars Tocai Friulano and Sauvignon Blanc, whereas *E. ambiguella* for the three red cultivars Carménère, Cabernet Sauvignon and Terrano. Therefore, it was shown for the first time that the relative preference for some cultivar was not the same for the two moth species.

### 4.2. Influence of Cultivar on Phenology of *L. botrana*

The grapevine cultivar influenced the phenology of *L. botrana* because, at the sampling time, larvae were older on Chardonnay than on the average of all cultivars together. Our data concerning the influence of cultivar on larval phenology are in agreement with the study reporting differences in adult emergence phenology [21]. In both studies, the development was the earliest on Chardonnay and the latest on Merlot. From a theoretical point of view, these differences in phenology may be due to a faster larval development or an earlier egg laying on some cultivars. The first hypothesis is supported by laboratory data indicating that larval development time is affected by the different food quality of the berries produced by each cultivar [19]. However, the maximum three-day difference in larval development time among cultivars reported in this last study does not seem sufficient to explain the differences in larval age composition observed in the field (data reported in [21] and in this study) because the interval between the two moultings is around a week. The differences in larval development time among cultivars could also be due to berry temperature, which is related to different amounts of sun exposure on bunches [20,36,37]. On the other hand, regarding the hypothesis that cultivar affects egg-laying periods, there is no evidence to suggest that females at the beginning of their oviposition period lay more eggs on berries of certain cultivars. However, this occurrence is known for the females of the overwintering generation that start to lay eggs on inflorescences of some cultivars before than on those of others [2,5,7].

The influence of grapevine cultivars on *L. botrana* phenology may have a practical consequence because a more widespread cultivation of some varieties could favour the selection of *L. botrana* populations suitable for developing an additional generation or at least increase the proportion of individuals of the penultimate generation that produces the last generation. Moreover, as suggested by another study [21], the influence of the grapevine cultivar on *L. botrana* development time should also be considered in the development of mathematical phenological models.

### 4.3. Influence of Bunch Traits on *L. botrana* Infestation and Phenology

Based on literature, bunch compactness has been associated with the highest infestation levels during the *L. botrana* carpophagous generations because it improved larval settlement [17]. Nevertheless, our results based on a high number of cultivars show that bunch compactness is not a decisive factor in determining differences in infestation levels. However, the cultivars with the highest and the lowest bunch compactness, i.e., Chardonnay and Merlot, were among the most and the least infested cultivars, respectively. Regardless, bunch compactness has an important role in the damage by vine moths because it is one of the factors that favours the spread of bunch rots from berries bored by larvae to contiguous un-bored berries [8]. Among other bunch traits considered, the average berry volume (i.e., berry size) was not a factor that favoured susceptibility, with Chardonnay and Tocai Friulano being the two most infested cultivars, but having the highest and the lowest berry volumes, respectively. Since some studies have reported that contact and volatile substances from berries are involved in cultivar susceptibility [4,14], the decisive factor that explains differences in population levels could be investigated in this research field. 

The positive relationship between bunch compactness and earliness in larval development observed in this study could be useful in explaining differences in larval phenology. Since in cultivars with very compact bunches the survival and settlement of the earliest larvae is greatest [17], we can hypothesize that on these cultivars larvae are on average older than those on cultivars whose bunches are loose in the earlier period of egg hatching. Additionally, the low larval settlement recorded at the berry set phenological growth stage [16] could support the occurrence of a lower larval settlement on cultivars closest to this stage during the egg-laying period. 

### 4.4. Relationship between First- and Second-Generation Infestations

The absence of a significant correlation between first- and second-generation infestations of vine moths within the same year indicates that, in the event of a high or low infestation during the first generation, we should not necessarily expect a high or low infestation during the second generation. Therefore, factors other than infestation potential seem more important in determining levels of infestation in the subsequent generation.

Cultivars with high levels of infestation during the second generation are not necessarily those with high infestation levels during the first generation. In our study, this phenomenon was particularly evident for Tocai Friulano, which was among the most infested cultivars during the second generation (this study) but among the least infested cultivars during the first generation [7]. The opposite occurrence was observed for Carménère. Obviously, the factors that increase the preference for a cultivar during the first generation (i.e., anthophagous generation) are different from those that make it preferable during the second generation. Differences in cultivar susceptibility have also been reported between second and third generations, with early harvest cultivars being relatively less infested during the third generation [11,24]. Regarding the early harvest cultivars, the preference by females to lay the eggs of the third and fourth generations on the second flowering berries [25,38] suggests that, when a cultivar is close to harvest, the first flowering berries are unattractive for egg laying. Indeed, on Chardonnay, females of the second generations laid a lower number of eggs per 100 berries on the first-flowering berries (0.46) than on the second-flowering berries (0.78 on those already developing specific colour and even 4.47 on those that were still green) (F. Pavan, unpublished data [39]).

## 5. Conclusions

Knowledge on cultivar susceptibility to vine moths has implications on the intensity of control measures to be adopted and the efficiency of sampling procedures. 

The earlier phenology of the *L. botrana* second generation associated with some cultivars (e.g., Chardonnay) must be taken into account because it determines both earlier phenology and greater amount of the third generation.

Based on larval infestation recorded on nine different cultivars, morphological bunch traits play no decisive role in determining susceptibility. Therefore, further research on contact and volatile substances originating from the berries is warranted.

## Figures and Tables

**Table 1:** Comparison of the average number of larval nests per year (four years) recorded on 100 bunches in the 10 cultivars. Different small letters among cultivars within each column indicate significant differences according to Tukey’s test at the 0.05 level.

Cultivar	Average ± Standard Deviation		
	Both Moth Species	*Lobesia botrana*	*Eupoecilia ambiguella*
Cabernet Sauvignon	21.3 ± 8.1 ab	13.2 ± 2.2 a	8.1 ± 6.0 a
Carménère	10.8 ± 8.8 a	6.1 ± 4.8 a	4.6 ± 4.1 a
Chardonnay	47.5 ± 27.4 b	36.0 ± 20.5 bc	11.5 ± 7.1 a
Merlot	20.8 ± 11.7 ab	14.4 ± 8.4 ab	6.4 ± 4.8 a
Refosco p.r.	24.0 ± 9.7 ab	18.8 ± 7.7 ab	5.2 ± 2.9 a
Rhine Riesling	21.0 ± 8.1 ab	16.2 ± 5.3 ab	4.8 ± 4.0 a
Sauvignon Blanc	23.5 ± 10.8 ab	19.3 ± 8.2 abc	4.2 ± 2.7 a
Terrano	20.5 ± 10.0 ab	11.1 ± 3.7 a	9.3 ± 6.5 a
Tocai Friulano	44.8 ± 20.4 b	38.0 ± 17.0 c	6.8 ± 3.9 a
Verduzzo Friulano	28.8 ± 11.5 ab	23.5 ± 9.0 abc	5.2 ± 3.3 a

**Table 2:** Cultivar bunch-traits recorded in coincidence with the *Lobesia botrana* egg-hatching peak of the second generation. Different small letters among cultivars within each column indicate significant differences according to Tukey’s test at the 0.05 level.

Cultivar	Average ± Standard Deviation			
	Compactness Index	Berry Number per Bunch	Total Berry Volume per Bunch (mL)	Average Berry Volume (mL)
Cabernet Sauvignon	0.31 ± 0.08 ab	100.9 ± 27.9 b	49.0 ± 17.6 ab	0.48 ± 0.06 c
Carménère	0.33 ± 0.08 bc	65.2 ± 16.7 a	40.9 ± 9.3 ab	0.63 ± 0.06 d
Chardonnay	0.41 ± 0.10 c	82.1 ± 17.2 ab	45.8 ± 13.7 ab	0.55 ± 0.06 cd
Merlot	0.20 ± 0.06 a	64.1 ± 24.7 a	34.0 ± 10.9 a	0.54 ± 0.06 cd
Refosco p.r.	0.36 ± 0.12 bc	106.5 ± 23.4 b	55.7 ± 10.5 b	0.54 ± 0.13 cd
Rhine Riesling	0.36 ± 0.07 bc	69.9 ± 21.5 a	32.5 ± 13.0 a	0.46 ± 0.06 bc
Sauvignon Blanc	0.33 ± 0.07 bc	104.7 ± 12.9 b	32.2 ± 10.3 a	0.30 ± 0.08 a
Tocai Friulano	0.27 ± 0.05 ab	103.2 ± 31.7 b	38.4 ± 12.1 ab	0.37 ± 0.06 ab
Verduzzo Friulano	0.26 ± 0.08 ab	94.7 ± 11.1 b	33.5 ± 11.0 a	0.35 ± 0.10 ab

**Table 3:** Linear regression between cultivar bunch traits and *Lobesia botrana* demographic parameters.

*Lobesia botrana* Demographic Parameters (Y)	Cultivar Bunch Traits (X)		
	Compactness Index	Total Berry Volume per Bunch	Average Berry Volume
Larval nests per 100 bunches (4-year average)	Y = 12.20 + 16.89X; *p* = 0.77; *R^2^* = 0.01	Y = 16.9 + 0.015X; *p* = 0.97; *R^2^* = 0.0002	Y = 33.0 − 33.0X; *p* = 0.30; *R*^2^ = 0.15
% 4th–6th instar larvae in 1997	Y = −61.0 +289.1X; *p* = 0.01; *R^2^* = 0.61	Y = 13.7 + 0.41X; *p* = 0.71; *R^2^* = 0.02	Y = 34.4 − 8.73X; *p* = 0.92; *R^2^* = 0.002
